# MATER protein expression and intracellular localization throughout folliculogenesis and preimplantation embryo development in the bovine

**DOI:** 10.1186/1471-213X-6-26

**Published:** 2006-06-06

**Authors:** Sophie Pennetier, Christine Perreau, Svetlana Uzbekova, Aurore Thélie, Bernadette Delaleu, Pascal Mermillod, Rozenn Dalbiès-Tran

**Affiliations:** 1 Physiologie de la Reproduction et des Comportements, UMR 6175 Institut National de la Recherche Agronomique/Centre National de la Recherche Scientifique/Université François Rabelais de Tours/Haras Nationaux, F-37380 Nouzilly, France

## Abstract

**Background:**

*Mater *(Maternal Antigen that Embryos Require), also known as *Nalp5* (NACHT, leucine rich repeat and PYD containing 5), is an oocyte-specific maternal effect gene required for early embryonic development beyond the two-cell stage in mouse. We previously characterized the bovine orthologue *MATER *as an oocyte marker gene in cattle, and this gene was recently assigned to a QTL region for reproductive traits.

**Results:**

Here we have analyzed gene expression during folliculogenesis and preimplantation embryo development. *In situ *hybridization and immunohistochemistry on bovine ovarian section revealed that both the transcript and protein are restricted to the oocyte from primary follicles onwards, and accumulate in the oocyte cytoplasm during follicle growth. In immature oocytes, cytoplasmic, and more precisely cytosolic localization of MATER was confirmed by immunohistochemistry coupled with confocal microscopy and immunogold electron microscopy. By real-time PCR, MATER messenger RNA was observed to decrease strongly during maturation, and progressively during the embryo cleavage stages; it was hardly detected in morulae and blastocysts. The protein persisted after fertilization up until the blastocyst stage, and was mostly degraded after hatching. A similar predominantly cytoplasmic localization was observed in blastomeres from embryos up to 8-cells, with an apparent concentration near the nuclear membrane.

**Conclusion:**

Altogether, these expression patterns are consistent with bovine MATER protein being an oocyte specific maternal effect factor as in mouse.

## Background

Preimplantation embryo development is largely dependent on maternal transcripts and proteins synthesized during oogenesis. Maternal factors are able to support the first cleavages, while blastocyst formation involves both maternal and embryonic factors. Over the last years, oocyte-restricted maternal effect genes have been the focus of much attention due to their specific expression profile and crucial function in early embryo development. They are predominantly expressed in oocyte, remain present in early embryos and then are degraded at the time of maternal-to-embryo transition (MET), without compensation by embryonic transcription. Functional studies based on knock-out mouse models have demonstrated their essential role in preimplantation embryo development, whereas functions in the oocyte itself have not been elucidated until this day.

*Mater *(Maternal Antigen that Embryos Require) is one such oocyte-specific maternal effect genes and was first identified in mouse [1,2]. The transcript and protein expression profiles have been investigated during oogenesis and preimplantation embryo development. *Mater *transcript and protein are first detected in oocyte from primary follicles and accumulate during oocyte growth. The transcript level decreases after fertilization as shown by ribonuclease protection assay or DNA array [3,4]). The protein remains abundant until the morula stage and is still present in blastocysts [3]. *Mater*-null females present a normal phenotype regarding folliculogenesis, ovulation and fertilization, but their embryos do not develop beyond the 2-cell stage coincident with the maternal-to-embryo transition. *Mater *precise function remains to be elucidated, although the global transcription decrease described in two-cell embryos lacking MATER may suggest a role in embryonic genome activation [2]. In our previous work, we identified the bovine orthologue of *Mater*. The longest open reading frame encodes a putative protein of 1098 amino acids (121 kDa). As its human counterpart, bovine MATER includes the 3 domains characteristics of the Nacht, Leucine rich repeat and Pyrin domain containing (NALP) family: a N-terminal Pyrin domain, followed by a NACHT domain and twelve C-terminal leucine rich repeats of the ribonuclease inhibitor subtype (LRR-RI). It is localized within a QTL region for reproductive traits [5]. *MATER *transcript pattern, i.e. its tissue distribution and its disappearance in blastocyst, appeared in agreement with bovine *MATER *also being an oocyte-specific maternal effect gene and suggested a conserved function as in mouse.

To reinforce this hypothesis, we needed to refine transcript expression during folliculogenesis, and mostly to characterize the expression and localization of the protein. In this study, we show that bovine MATER transcript and protein are expressed in the oocyte as early as the primary follicle stage and accumulate during folliculogenesis. The protein localizes in the cytosol of immature oocytes. It remains abundant in the cytoplasm of preimplantation embryos until the blastocyst stage and is mostly degraded after hatching.

## Results

### Antibody characterization

**Figure 1:**
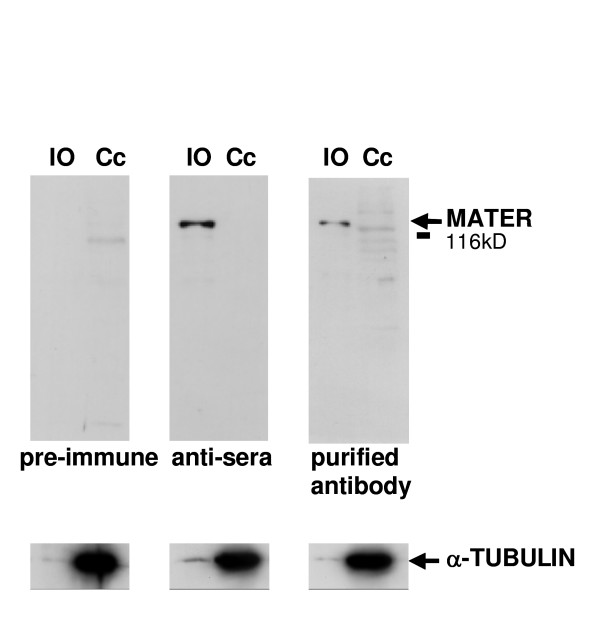
**Characterization of anti-MATER serum and purified antibody by Western blot**. Detection of MATER in immature oocytes (IO) but not in cumulus cells (Cc) with anti-peptide serum or purified antibody, nor with the preimmune serum. Molecular weight is indicated on the left.

First, we checked our antipeptide serum and purified antibody for sensitivity and specificity by western blotting (Fig. 1). Under normal exposure, a single intense band was detected at the MATER protein predicted molecular weight (121 kDa) with as few as 10 oocytes. No such band was detected in a protein extract from cumulus cells in huge excess (see the amount of TUBULIN), although faint bands were observed at various molecular weights. Specificity was further confirmed by the absence of MATER signal in oocytes using preimmune serum.

### MATER expression throughout folliculogenesis

**Figure 2:**
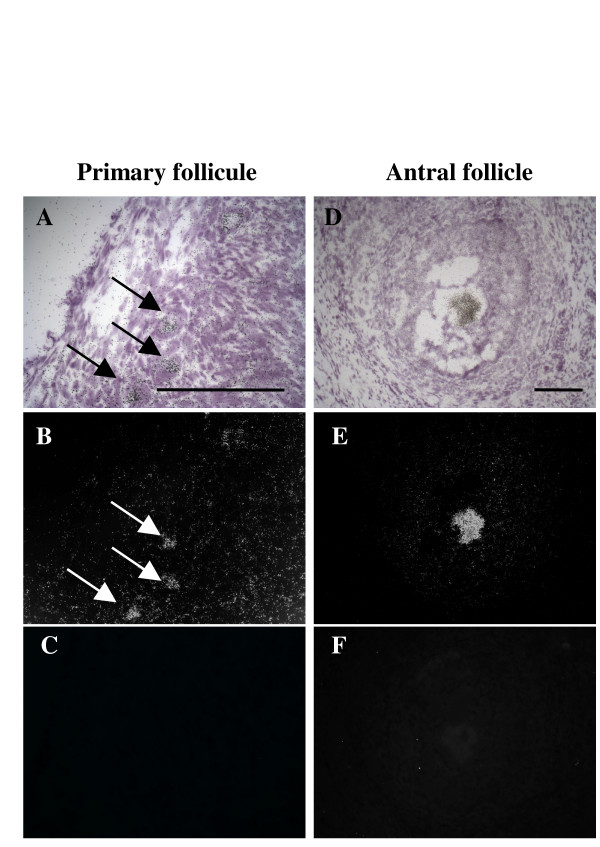
**Ovarian localization of bovine *MATER *transcript by *in situ *hybridization**. Bright field (A, D) and dark field (B, C, E, F) photomicrographs of adjacent ovarian sections showing primary follicles (A-C, arrows), and an antral follicle (D-F) hybridized with either antisense (A, B, D, E) or sense (C, F) *MATER*-[^35^S] riboprobes. Scale bar: 100 μm.

The expression of *MATER *transcript during folliculogenesis was analysed using *in situ *hybridization onto ovarian sections. As previously described [6], *MATER *expression was restricted to the oocyte within bovine ovary. In this experiment, a signal could be detected in oocyte as early as the primary follicle stage and at a higher level in oocyte from antral follicle. Hybridization with the corresponding sense probe was used as negative control (Fig. 2).

**Figure 3:**
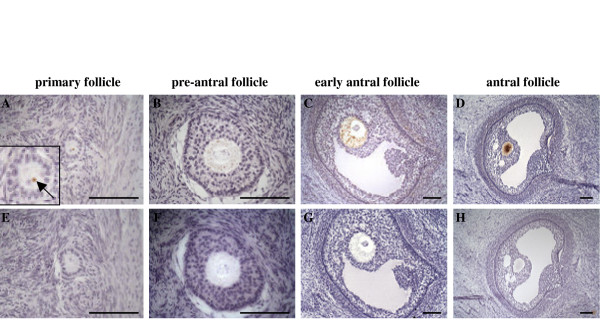
**Ovarian localization of bovine MATER protein by immunohistochemistry**. Adjacent ovarian sections showing primary follicle (A, E), pre-antral follicle (B, F), early antral follicle (C, G) and over 1 mm antral follicle (D, H) incubated with either anti-MATER serum (A-D) or preimmune serum (E-H) followed by biotinylated horse anti-rabbit IgG antibody. Scale bar: 100 μm. Inset in A is the same oocyte shown at higher magnification.

To further characterize *MATER *gene expression during folliculogenesis, we studied its expression pattern at the protein level. Using anti-MATER serum, the protein was exclusively detected in oocyte and no staining was observed in granulosa or theca cells. MATER was detected in oocyte of primary follicles at a low level, with a very distinctive pattern: the staining localized to a single dot (Fig. 3A) which was specifically and reproductively observed. As folliculogenesis proceeds, staining intensity increases in oocyte from preantral to small antral and antral follicles (Fig. 3B–D), suggesting an accumulation of the protein. Interestingly, on sections that went through the nucleus in pre-antral and early antral follicles, the protein appeared excluded from the germinal vesicle (Fig. 3B, C).

### MATER expression during preimplantation embryo development

**Figure 4:**
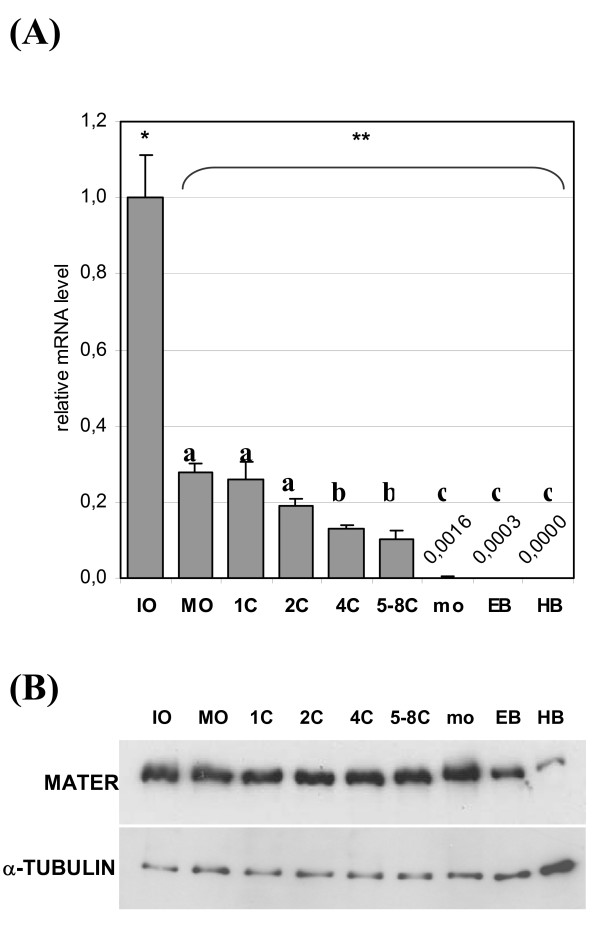
**Expression of bovine *MATER *in oocytes and preimplantation embryos**. Developmental stages are immature oocytes (IO), *in vitro *matured oocytes (MO), *in vitro *cultured zygote (1C), 2-, 4-, 5- to 8- cell embryos, morula (mo), expanded blastocysts (EB) and hatched blastocysts (HB). (A) Quantification of the messenger RNA using real-time PCR. Relative level to immature oocytes is shown (mean ± SEM). In late stage embryos, transcript level is indicated but too low to be seen on the histogram. Different superscripts indicate a significant difference (P ≤ 0.05). (B) Detection of the protein by Western blot; α-TUBULIN is shown as a positive control.

Evolution of MATER messenger RNAs during during oocyte maturation, fertilization and preimplantation embryo development was followed by real time PCR. Triplicate reactions were run onto four independent samples at each stage. The level relative to immature oocytes is shown (Fig. 4A). Detection level decreased by 72% during maturation, then remained stable during fertilization. It decreased progressively during the cleavage stages, up to 10% of its initial level in 5 to 8 cell-embryos. In morulae and blastocysts, amplicons were produced only in one or two samples; the level of polyadenylated transcript was less than 0.5% of its initial value. Western blotting was then performed to characterize MATER protein expression (Fig. 4B). MATER was abundant during oocyte maturation, and after fertilization until the expanded blastocyst stage. Most protein was then degraded after hatching. This pattern was reproducibly observed with two independent oocyte and embryo collections. By contrast, our positive control β-TUBULIN increased in hatched blastocysts (Fig. 4B).

### MATER protein localization in oocyte and preimplantation embryos

**Figure 5:**
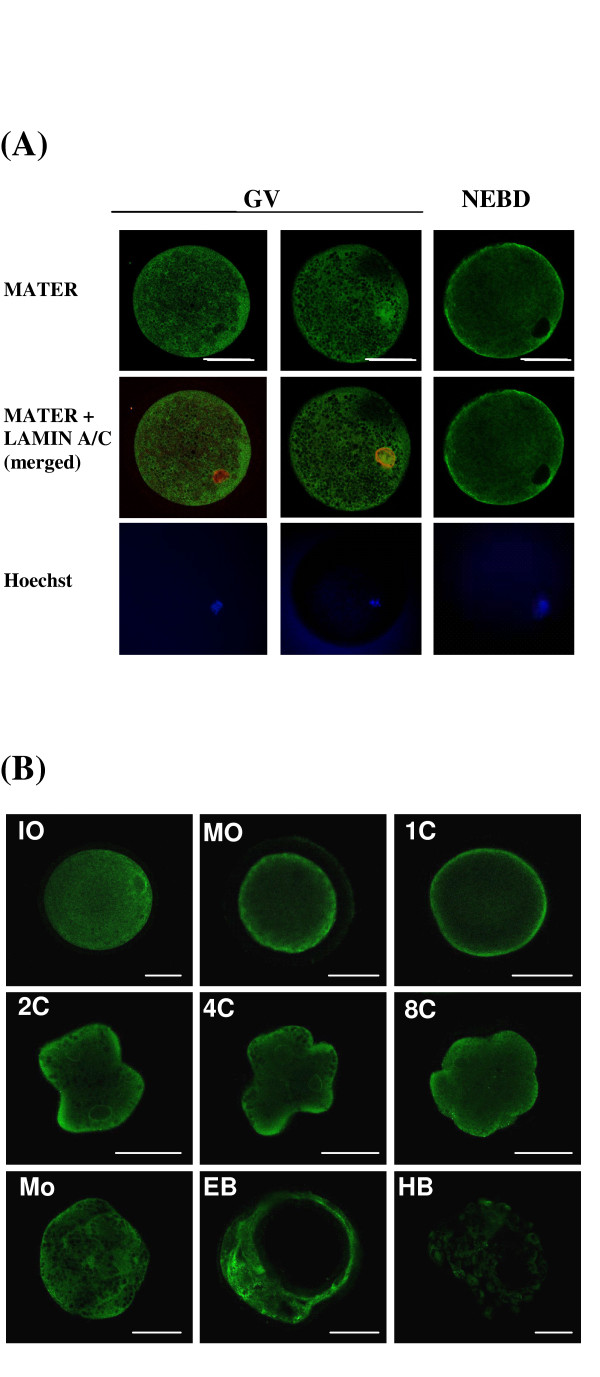
**Localization of MATER protein in bovine oocytes and preimplantation embryos by confocal microscopy**. Oocytes and embryos were incubated with anti-MATER peptide purified antibody (1:250) and revealed by green staining. Scale bar: 50 μm. (A) Oocytes at GV or NEBD stage. Lamins A/C are stained in red (middle row). Chromatin is stained in blue with Hoechst 33258 (lower row). (B) immature oocytes (IO), *in vitro *matured oocytes (MO), *in vitro *cultured zygote (1C), 2-, 4-, 8- cell embryos, morula (mo), expanded blastocysts (EB) and hatched blastocysts (HB).

Immunohistochemistry onto ovary sections suggested that MATER could be predominantly cytoplasmic. To refine protein localization, we performed confocal microscopy analysis on oocytes from 3 to 8 mm follicles and preimplantation embryos, after immunofluorescent staining using purified antipeptide (Fig. 5). Firstly, we observed oocytes at germinal vesicle (GV) and nuclear envelope break down (NEBD) stages, as discriminated by LAMIN A/C staining (Fig. 5A). In most GV oocytes, MATER protein was predominantly localized in the cytoplasm (Fig. 5A, left panel); however, a few oocytes (less than 10%) presented a distinct pattern, with staining within both the cytoplasm and the nucleus (Fig. 5A, middle panel). In oocytes characterized by degradation of LAMIN A/C (Fig. 5A, right panel), MATER was distributed throughout the oocyte, only excluded from the chromatin area as revealed by Hoechst staining. Then we analysed mature oocytes, zygotes and preimplantation embryos (Fig. 5B). MATER was predominantly detected in the cytoplasm, with the protein apparently concentrating in the cortical region of mature oocytes, zygotes and embryos at least until the 8-cell stage. In zygotes to 8-cell embryos, increased staining around the chromatin area (as revealed by Hoechst staining, not shown) suggested that MATER also accumulated around the nuclear membrane. In expanded blastocysts, MATER was distributed evenly between inner cell mass and trophectoderm. A dramatic decrease was observed in hatched blastocysts, as previously observed by Western blotting. As negative control, no staining was revealed using rabbit IgG as primary antibody (not shown).

**Figure 6:**
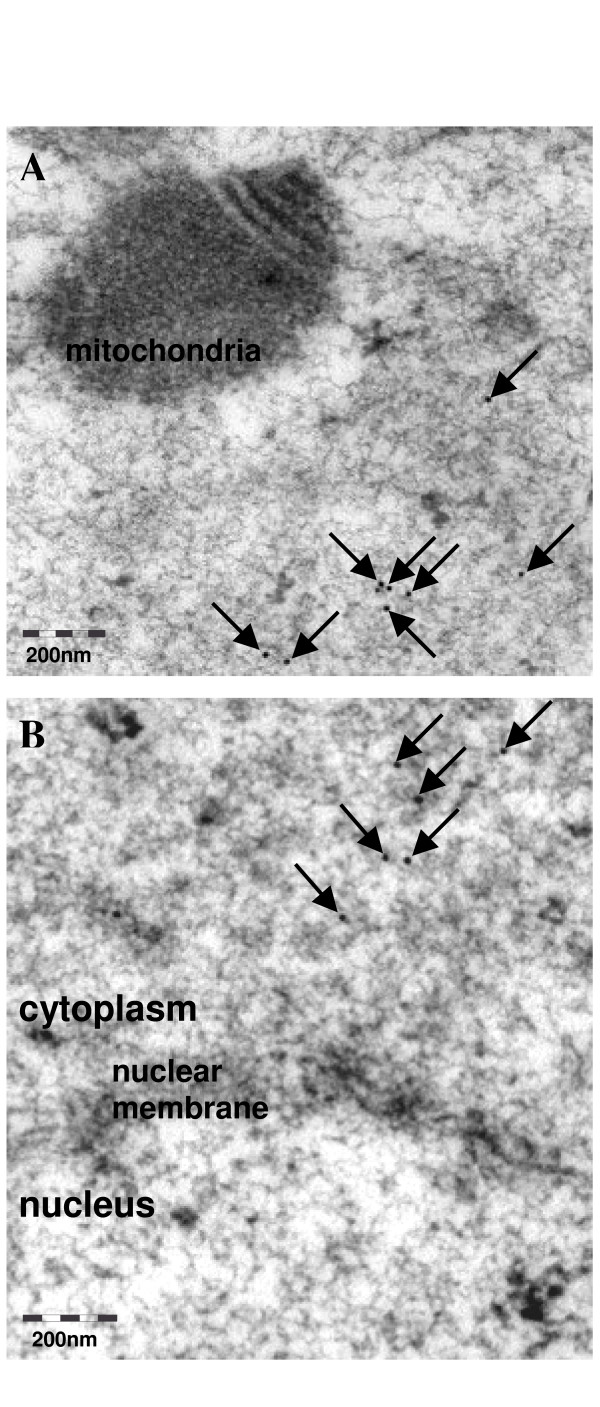
**Subcellular localization of MATER protein in bovine immature oocytes by transmission electron microscopy**. Immunogold particles are pointed at by arrows. (A) region including a mitochondria (B) a detail of the nuclear membrane, separating the nucleus (below) and the cytoplasm (above).

To refine MATER protein subcellular localization, transmission electron microscopy analysis was performed on ultrathin sections of immature oocytes using purified antipeptide. Immunogold particules were diffusely present in the cytosol at a low density. MATER protein was not detected in the nucleus, nor in organelles including mitochondria (Fig. 6). As a negative control, no particles were observed when omitting the primary antibody (not shown).

## Discussion

In this study, we characterized the bovine *MATER *gene and its expression throughout folliculogenesis and in vitro preimplantation embryo development. We demonstrated that both the transcript and protein were present in oocyte from primary follicle and accumulated thereafter. During preimplantation embryo development, MATER messengers were hardly detected after the morula stage, while the protein remained present until the blastocyst stage before rapid degradation after hatching. In immature oocytes and in embryos, the protein was predominantly cytoplasmic.

### MATER gene expression throughout bovine folliculogenesis and preimplantation embryo development

In a previous study, we demonstrated that *MATER *transcript was restricted to the oocyte within bovine ovary [6]. Here, we have refined our expression analysis. By *in situ *hybridization and immunohistochemistry experiments, we detected *MATER *transcript and protein in oocytes as early as the primary follicle stage (Fig. 2 and 3), as previously described in mouse [1,3]. This shows that at least some transcripts support immediate translation, but it does not exclude that others are stored within the oocyte cytoplasm. Such an early protein synthesis may appear premature since functional studies did not reveal a deleterious effect of *Mater *absence at this early stage, but rather in the 2-cell embryo [2]. Indeed, ZAR1, another mouse oocyte-specific maternal effect protein, was also detected in mouse primary follicles [7]. Several scenarios may reconcile these apparent discrepancies. The first hypothesis is that MATER indeed plays a role in the oocyte, but that another gene may be redundant at this stage, at least in *Mater*-null females. Obvious candidates are genes of the NALP family, and especially oocyte restricted members such as *NALP9 *in bovine [8] or the *Nalp9 *or *Nalp4 *genes in mouse [9,10]. To our knowledge, there are no functional data on these genes from knock-out models or other targeted inhibition experiments. This early expression may also be indicative of an upstream role of MATER in a cascade of events, as demonstrated for the mouse oocyte-specific homeobox gene *Nobox *[11]. Alternatively, the protein may be synthesized despite not being required at this stage, simply because specifically repressing the gene would be more complex for the cell than support minimal expression. Overall, we observed that immunostaining of MATER protein increased in parallel with follicle development, from primary to large antral. This must involve a growing level of translation; in addition, it is possible that once synthesized, the protein remains stable for a long period of time and persists in the oocyte during folliculogenesis. Neo-synthesized proteins would then add to those already present, and early synthesis would allow for the accumulation of a largest stockpile of MATER.

By real-time PCR, bovine MATER transcripts showed a massive deadenylation and/or degradation during maturation (Fig 4A). Following fertilization, the messengers slowly decreased in embryos until the 5- to 8-cell stage; it was hardly detected in morulae and blastocysts, confirming our previous report [6]. We did not evidence neo-synthesis or polyadenylation of MATER transcripts neither transiently nor after the MET. By contrast, we have demonstrated by Western-blot and immunofluorescence coupled to confocal microscopy that the protein is present at all stages, and displays a sharp decrease only after hatching (Fig. 4 and 5). These distinct patterns for the transcript and the protein provide valuable information. First, later persistence of the protein as compared to the messenger is consistent with MATER being a fairly stable protein. This, together with the accumulation of MATER during folliculogenesis, suggests that at least some protein detected in early embryos is of maternal origin. Second, degradation of MATER protein after hatching appears temporally controlled and substrate specific, since most proteins are rather synthesized at this stage following the MET. The bovine protein in fact persists longer than its mouse counterpart, which was mostly degraded by the expanded blastocyst stage. This is consistent with active degradation of MATER involving factors synthesized from embryonic transcripts in both species. The ubiquitin-proteasome pathway is one possible mechanism, and ubiquitin transcripts were actually shown to increase in bovine blastocysts [12]. On the other hand, ubiquitin cross-reactive structures were detected preferentially in the trophoblast over the the inner cell mass [13] whereas here we observe a simultaneous degradation of MATER in both structures at hatching (Fig. 5). Overall, the pattern of expression of bovine MATER, i.e. non reactivation at MET and degradation of the protein before implantation, supports the hypothesis that it is a maternal effect gene as its mouse orthologue.

### MATER protein intracellular localization in bovine oocytes and preimplantation embryos

Data from immunohistochemistry on ovary sections suggested that MATER protein was stored in the cytoplasm of oocyte during folliculogenesis (Fig. 3B, C). Intracellular localization of MATER was further investigated in oocytes and early embryos by immunofluorescence coupled to confocal microscopy analysis (Fig. 5). In most immature oocytes, MATER was predominantly localized in the cytoplasm with an accumulation around the nuclear membrane and was hardly detected in the germinal vesicle. A few oocytes displayed a different pattern of protein distribution, where MATER was detected both in the cytoplasm and the germinal vesicle (Fig. 5A). After NEBD, the protein was uniformly distributed throughout the cytoplasm and was only excluded from the chromatin area (Fig. 5A). Such transient nuclear localization of MATER was not described in mouse oocytes. We propose two hypotheses consistent with our observations. It may be that MATER passively diffuses to the germinal vesicle as the nuclear membrane starts to disaggregate, even though lamins are still detected. Alternatively, we can speculate that MATER actively translocates to the nucleus just before NEBD. MATER sequence does not display a nuclear localization signal as analyzed by the PredictNLS server [14,15]. But neither was such signal identified in the mouse oocyte specific variant of DNA (cytosine-5)-methyl-transferase 1 (Dnmt1o), whose temporarily controlled nucleocytoplasmic trafficking was nevertheless demonstrated: this protein localizes to the cytoplasm of oocytes and preimplantation embryos with exclusion from all nuclei except those of 8-cell embryos [16]. MATER might be involved in the short burst of transcription that has been suggested to occur in bovine oocyte-cumulus complexes just before chromatin condenses [17-19]

Then, we followed MATER localization after oocyte maturation, fertilization and during preimplantation embryo development (Fig. 5B). Localization was predominantly cytoplasmic in embryos at least until the 8 cell stage. We noticed that the protein appeared to concentrate in the cortical region of mature oocytes and embryos up to 8-cells, as previously observed in mouse preimplantation embryos [3]. A similar peripheral concentration had also been reported for the ovary-specific 2',5'-oligoadenylate synthetase-like protein (OAS1D) in metaphase II oocytes [20], and for DNMT1o in mature oocytes, 2- and 4-cell embryos [16]. The authors suggested that DNMT1o may be sequestered near the plasma membrane through interaction with annexin V, a calcium-sensitive phospholipid binding protein [16]. MATER includes protein-protein interacting domains and identifying potential partners may help elucidating this intriguing distribution.

In GV oocytes, MATER localization was refined by immunogold coupled to transmission electron microscopy analysis. The protein was detected in the cytosol but not in organelles (Fig. 6). This pattern differs from MATER distribution in mouse, where the protein was detected in the nucleus close to nuclear pores and in mitochondria [3].

Based on the presence of a leucine-rich repreat domain homologous to a porcine ribonuclease inhibitor, mouse MATER has been suggested to play a role in mRNA stability. Altogether, our observations on bovine MATER distribution are consistent with this hypothesis. In oocytes, MATER would stabilize transcripts from the moment when they are exported from the nucleus and during their storage in the oocyte cytoplasm up until the time when there are recruited for translation in the oocyte or after fertilization. Through a similar peri-nuclear accumulation in early embryos, MATER could protect the rare transcripts produced from the embryonic genome at these stages. Finally, MATER is degraded after hatching, once the embryo synthesizes large amount of messengers and proteins. This model is consistent with the phenotype of embryos from *Mater *null female mice: transcripts required for development beyond the 2-cell stage (e.g. in genome activation) would not be stabilized, resulting in developmental block.

## Conclusion

We have demonstrated that MATER transcript and protein are specifically expressed in oocyte from the primary follicle onwards in bovine ovary. The protein is stored in the cytoplasm of growing oocytes and persists during maturation, fertilization and in embryos until the expanded blastocyst stage. It is mostly degraded after hatching. Therefore *MATER *transcript and protein expression patterns are consistent with a maternal effect function conserved in bovine. Challenging functional approaches are now required to confirm this hypothesis.

## Methods

### Oocyte collection and in vitro embryo production

Ovaries from adult cows (unless otherwise specified) were collected at a slaughterhouse and the oocyte-cumulus complexes were aspirated from 3–6 mm follicles, selected based on morphological criteria and washed in saline solution (for details see [21]). Denuded immature and *in vitro *matured oocytes were obtained as previously described [22]. Briefly, oocyte-cumulus complexes were denuded by mechanical treatment either before or after *in vitro *maturation in TCM199 (Sigma, Saint Quentin Fallavier, France) supplemented with 10 ng/ml epidermal growth factor and 10% fetal calf serum for 24 hr at 39°C in water-saturated air with 5% carbon dioxide. For *in vitro *fertilization, groups of 50 intact *in vitro *matured oocyte-cumulus complexes were transferred into 500 μl fertilization medium containing 10^6 ^motile spermatozoa. 24 hr later, presumptive zygotes were denuded. Groups of 25 zygotes were transferred into 25 μl droplets (under paraffin oil) of modified synthetic oviduct fluid supplemented with 5% fetal calf serum. Embryos were cultured at 39°C in a water-saturated atmosphere with 5% CO_2_/5% O_2_/90% N_2_. Groups of 10 embryos were collected over preimplantation development period: zygote and 2-cell embryos (day 2), 4-cell, 5 to 8-cell embryos (day 3), morulae (day 5), expanded blastocysts (day 6) and hatched blastocysts (day 8). All oocyte and embryo samples were stored frozen at -80°C. Two and four independent sets of oocytes and embryos were collected for immunological and real-time PCR analyses respectively.

### In situ hybridization

Ovaries from 6 month old calves were embedded in Tissue-Tek medium (Sekura, Bayer Diagnostics, France), frozen in liquid nitrogen and then serially sectioned (10 μm) with cryostat. The sections were fixed in 4% paraformaldehyde. A 391 bp *MATER *PCR fragment (corresponding to nt 2819–3209 of the coding sequence) was subcloned in Dual Promoter pCRII plasmid (Invitrogen, Cergy-Pontoise, France). Sense and antisense probes were generated using Riboprobe combined system SP6/T7 (Promega, Charbonnières, France) and labelled with [^35^S]-UTP. Hybridization and washed were performed as described elsewhere [23]. Sections were counterstained with hematoxylin.

### Real-time PCR

After adding 10 pg of luciferase mRNA (Promega) as an exogenous standard, total DNAse-treated RNA was extracted from 4 independent pools of 10 oocytes or embryos at each stage using the Picopure RNA isolation kit (Alphelys, Plaisir, France). Reverse transcription was performed using oligo(dT)_15 _primers (Promega) during 50 min at 37°C by mouse Moloney leukaemia virus reverse transcriptase (Invitrogen, cergy Pontoise, France). Target cDNA were then quantified by real-time PCR using iQ SYBR green supermix (Bio-Rad, Marnes la Coquette, France) in a MyCycler system (Bio-Rad) using specific primers for *MATER *(forward 5'-GCTGGAGGCGTGTGGACTG; reverse 5'-GGTCTGTAGATTAGAGGTGGGATGC) and *luciferase *(forward 5'-TCATTCTTCGCCAAAAGCACTCTG; reverse 5'-AGCCCATATCCTTGTCGTATCCC). A 3-step protocol was repeated for 40 cycles (95°C for 30 sec, 60°C for 30 sec, 72°C for 20 sec), followed by acquisition of the melting curve. cDNA amount equivalent to 0.05 oocyte or embryo was used in triplicate reactions. The standard curve was deduced from five serial dilutions (100 fg to 0.01 fg) of a plasmid including the target sequence included in each run. In each sample, the median value of PCR triplicates was considered (0 when not detected), and MATER data was normalized with exogenous luciferase to correct for loss of RNA; the MATER/luciferase value was then compared to this same ratio in immature oocytes (mean of four oocyte collections). Data are presented as mean ± SEM. After analysis of variance, first between immature oocytes and other stages, then between stages starting from mature oocyte onwards, differences were considered statistically significant at the 95% confidence level (P ≤ 0,05) using Tukey comparison test.

### Immunohistochemistry

A keyhole limpet hemocyanin (KLH)-coupled MATER peptide (C terminus, aa 1084–1098) was used to immunize rabbits to produce the primary antibody (Eurogentec, Angers, France). Bovine ovaries were fixed in Bouin solution (50% saturated picric acid, 3.7% formaldehyde, 5% acetic acid), embedded in paraffin and serially sectioned (10 μm). The ovarian sections were immersed in antigen unmasking solution (Abcys, Paris, France) and warmed for 6 min in a microwave. After removing endogenous peroxydase activity in 0,3% H_2_O_2 _and blocking with horse serum, ovarian sections were incubated either with primary anti-MATER serum or rabbit preimmune serum (1:10000) overnight at 4°C, followed by incubation for 1 hr at room temperature with secondary biotinylated horse anti-mouse/rabbit/goat IgG antibody (1:200) (Abcys). Avidin, biotinylated horseradish peroxydase (HRP) and diamino-benzidene (DAB) were applied according to the manufacturer's instructions using Vectastain elite ABC kit (Abcys). Sections were counterstained with hematoxylin.

### Western blot

Pools of 10 oocytes or embryos, or cumulus cells, were frozen and thawed three times, and then the proteins were separated on 7.5 or 10% SDS-PAGE gels and transferred onto a nitrocellulose membrane. After blocking with 5% dry milk in tris buffered saline, the blot was incubated with anti-MATER serum (1:10000) or pre-immune serum (1:10000) or purified antibody (1:2000) overnight at 4°C under agitation, and subsequently with secondary HRP-conjugated goat anti-rabbit IgG antibody (1:5000) for 1 hr 30 min at room. The signal was detected using an enhanced chemiluminescence kit according to the manufacturer's instructions (Amersham Biosciences, Orsay, France). The membranes were stripped and blotted with an anti-α-TUBULIN monoclonal antibody (Sigma). Experiment was performed on two independent oocyte and embryo collections.

### Confocal scanning laser microscopy

Oocytes and embryos were fixed for 20 min in 4% paraformaldehyde. After treatment in phosphate buffered saline 1X/Triton 0.2% followed by blocking in 5% goat serum, they were subsequently incubated with either rabbit anti-MATER antibody purified onto a peptide affinity column by the manufacturer (1:250) or rabbit IgG overnight at 4°C, and then for 1 hr at room temperature with secondary goat anti-rabbit IgG antibody conjugated to fluoprobe 488 (1:100) or fluorescein isothiocyanate (1:400) for immature oocytes (both from Interchim, Montluçon, France). For LAMIN staining, immature oocytes were incubated with mouse anti-LAMIN A/C antibody (1:200; Ozyme, Saint Quentin Yvelines, France) for 2 hr at room temperature and with secondary Texas Red-conjugated goat anti-mouse antibody (1:400; Sigma), for 1 hr at room temperature. Chromatin was stained using Hoechst 33258 (Sigma). Mowiol was used as mounting medium.

### Transmission electron microscopy (TEM)

Immature oocytes were fixed in 4% paraformaldehyde for 20 min at 4°C. Subsequently, they were washed in phosphate buffer 0.1 M, incubated in ammonium chloride 0.05 M in phosphate buffer for 30 min at room temperature and washed in phosphate buffer. Then, oocytes were dehydrated in ethanol and embedded in LRWhite resin (London Resin Company, Theale, England). Ultra-thin sections (80 nm) were placed on 200 mesh formvar-coated nickel grids and blocked in 10% goat serum and 1% BSA. The grids were then incubated with purified rabbit anti-MATER antibody (1:1000) overnight at 4°C, washed with PBS/0,1% BSA/1% goat serum and incubated with colloidal gold-labelled (18 nm) secondary goat anti-rabbit antibody (1:10, (Immunotech, Marseille, France) for 2 hr at room temperature. After several washing in PBS and deionised water, sections were counterstained with 4% uranyl acetate and lead citrate. Specificity of immunostaining was checked by incubation of sections with PBS/0,1% BSA/1% goat serum. Sections were observed with a CM10 electron microscope (Philips, Eindhoven, Netherlands).

## Authors' contributions

SP participated in experimental design and carried out most experimental work. CP collected biological material, carried out in vitro embryo production, and participated in immunohistochemistry. BD carried out the TEM experiments. SU was involved in biological sample collection, Western blot, and provided input in writing the manuscript. AT performed oocyte/embryo collection and real-time PCR. PM provided expertise in experimental design and analysis. RDT designed and supervised the study, and participated in experimental work (oocyte collection, Western blot). SP and RDT wrote the manuscript.
